# Associations between oxidation balance score and abdominal aortic calcification, and the mediating role of glycohemoglobin: a nationally representative cross-sectional study from NHANES

**DOI:** 10.3389/fnut.2025.1469449

**Published:** 2025-01-20

**Authors:** Heqian Liu, Yifei Wu, Zhenyu Liu, Hongzhi Ren, Ya Wu, Yong Liu

**Affiliations:** ^1^Department of Vascular Surgery, The Affiliated Hospital of Southwest Medical University, Luzhou, China; ^2^Metabolic Vascular Disease Key Laboratory of Sichuan Province, The Affiliated Hospital of Southwest Medical University, Luzhou, China; ^3^Key Laboratory of Medical Electrophysiology, Ministry of Education and Medical Electrophysiological Key Laboratory of Sichuan Province (Collaborative Innovation Center for Prevention of Cardiovascular Diseases), Institute of Cardiovascular Research, Southwest Medical University, Luzhou, China

**Keywords:** abdominal aortic calcification, oxidation balance score, postmenopausal women, glycohemoglobin, mediating role

## Abstract

**Background:**

Abdominal aortic calcification (AAC) is prevalent among middle-aged and elderly populations, elevating the risk of cardiovascular and cerebrovascular events. Leveraging data from the National Health and Nutrition Examination Survey (NHANES), we conducted a nationally representative cross-sectional study. Our aim was to evaluate whether subjective interventions could influence AAC scores by modifying the antioxidant/pro-oxidant status of individuals and to investigate the role of glycohemoglobin in this relationship.

**Methods:**

The study analyzed data from 1,600 U.S. adults. The study used oxidation balance score (OBS) as an exposure variable derived from 16 dietary and 4 lifestyle factors, as well as glycohemoglobin obtained from blood tests. The main outcome measure was AAC, which was evaluated by dual-energy X-ray absorption and quantified by Kauppila scoring system.

**Results:**

The mean (SD) age of the 1,600 patients was 56.53 (10.90) years, with a female predominance (50.22%). According to weighted linear regressions not adjusted for covariates, the AAC scores were lower in the third and fourth quartile groups of OBS than in the first quartile group of OBS (Q3: coefficients [coef], −0.92 [95% CI, −1.64 to-0.20], *p* = 0.017; Q4: coefficients [coef], −0.97 [95% CI, −1.86 ~ −0.08; *p* < 0.035]). According to the weighted linear regression subgroup analyses, there were no significant OBS-AAC correlations among males (*p* > 0.05), but there were significant correlations among females (*p* < 0.05). Smooth fitting curves showed a more significant trend of change in the negative correlation between OBS and AAC scores in postmenopausal women than in premenopausal women. In further mediation analyses, glycohemoglobin was identified as a mediator of the relationship between the oxidative balance score and AAC.

**Conclusion:**

This study revealed a significant negative correlation between OBS and AAC scores, particularly in postmenopausal women. The negative correlation between OBS and AAC is partly mediated by glycohemoglobin.

## Introduction

Vascular calcification (VC) is a common pathological process in the human cardiovascular system that is most commonly observed in renal failure, atherosclerosis, diabetes mellitus (DM), and aging and can lead to stiffening of the vascular wall and reduced compliance, leading to left ventricular hypertrophy, heart failure, thrombosis, plaque rupture, and other adverse events with high rates of death and disability (1). VC is often concentrated in the abdominal aortic region, subsequently giving rise to abdominal aortic calcification (AAC). It has been shown that widespread AAC significantly interferes with the Windkessel effect, thus hampering the normal physiologic function of the aorta (2). In addition, several studies have shown that AAC is closely associated with the occurrence of cardiovascular events related to coronary heart disease or chronic kidney disease (3, 4). However, the mechanism of vascular calcification is complex, and there is a lack of effective therapeutic strategies for reversing or curing vascular calcification in the clinic (5). Therefore, exploring potential interventions that are beneficial for the prevention of AAC could contribute to the early prevention of future cardiovascular-related adverse events.

Oxidative stress (OS) is considered to be an extremely important factor in the progression of vascular calcification (6). Excessive reactive oxygen species (ROS) generated by oxidative stress are key mediators of a variety of mechanisms that promote vascular calcification, with effects on phosphate homeostasis *in vivo*, as well as contributing to the transdifferentiation of vascular smooth muscle cells and, to some extent, triggering and promoting inflammatory responses (8). Therefore, antioxidants may be considered a therapeutically effective option for vascular calcification. Under normal physiological conditions, the oxidative and antioxidant systems are in equilibrium, and the oxidative/antioxidant balance is influenced by numerous factors, including physical activity, smoking, alcohol consumption, obesity, and various dietary components (7). The oxidation balance score (OBS) comprehensively assesses an individual’s anti/promotional oxidant exposure by calculating 16 anti/promotional dietary elements and 4 anti/promotional lifestyle elements in each individual’s daily life (9), where a high score represents predominant antioxidant exposure. Previous studies have shown a strong association between OBS and certain diseases, especially some chronic diseases, such as osteoporosis (10), hypertension (11), and even gastric cancer (12). However, to date, no studies have evaluated the relationship between AAC and OBS. In our study, we investigated the association between AAC and OBS and tried to look for mediating factors. Therefore, this cross-sectional survey used information from the National Health and Nutrition Examination Survey (NHANES) to examine the potential relationship between AAC and OBS in middle-aged and elderly individuals. These findings may provide new clinical strategies for the prevention and management of AAC and severe AAC.

## Methods

### Data sources and participant populations

The NHANES is a cross-sectional survey based on a probability-based multistage sampling design whose primary purpose is to collect basic data on the health status of all noninstitutionalized U.S. civilians. All the data are publicly available and can be accessed through the NHANES website.^1^ The survey is administered under the leadership of the National Center for Health Statistics (NCHS). The NHANES protocol was approved by the Research Ethics Review Board at the National Center for Health Statistics, and written informed consent was obtained from all participants. To date, the NHANES database contains only AAC scores for 2013–2014; therefore, the study analyzing the association between OBS and AAC in this cross-sectional survey was conducted using NHANES data for 2013–2014 and met the criteria for Strengthening the Reporting of Observational Studies in Epidemiology (STROBE) (13).

**Figure 1:**
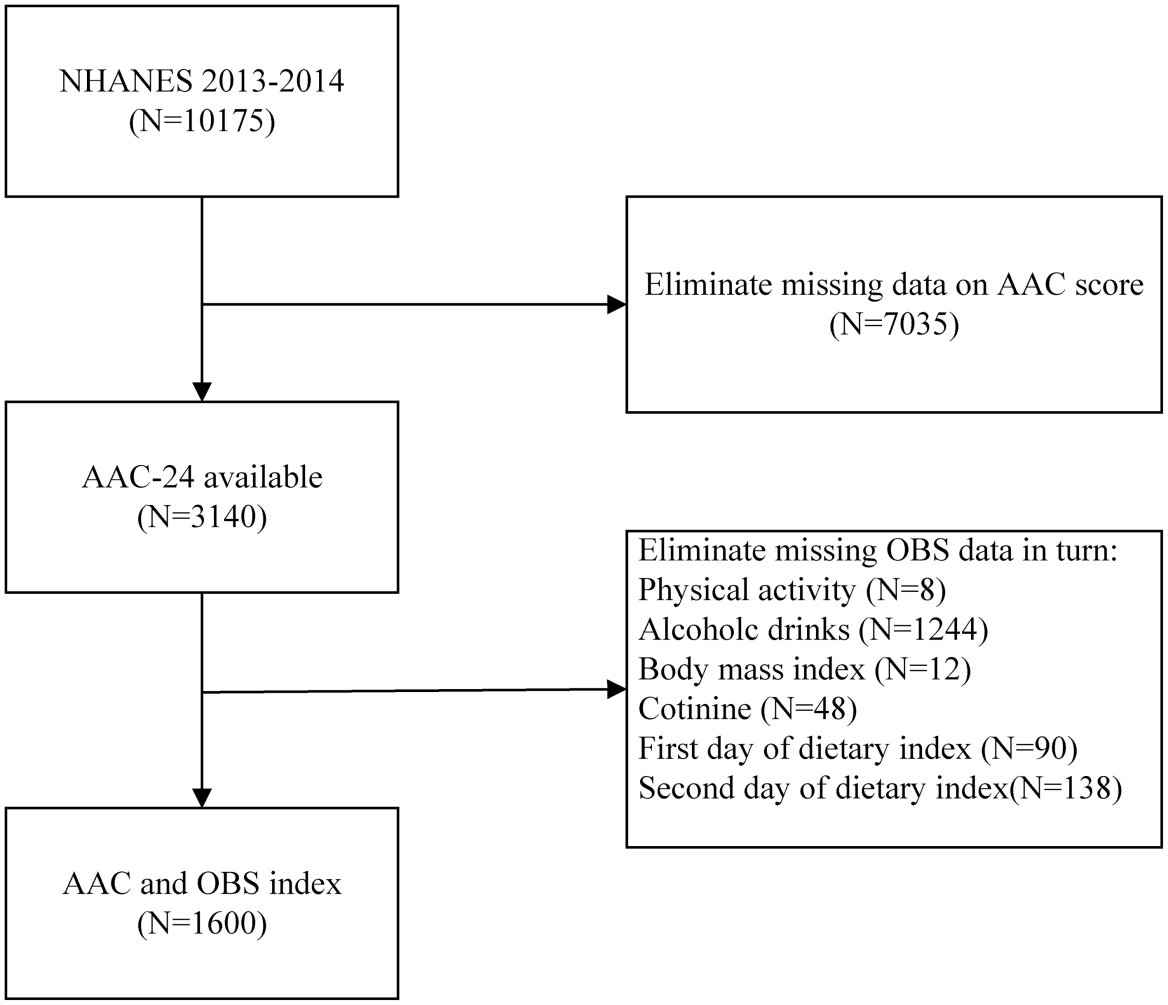
Flowchart of the sample selection process. AAC, abdominal aortic calcification.

AAC scores were determined by performing dual-energy X-ray absorptiometer (DXA) scans on subjects who were eligible to participate in dual-energy radiography scans of the thoracolumbar spine in 2013–2014, with lateral dual-energy radiography scans of the thoracolumbar spine at the NHANES Mobile Screening Center, with the following exclusionary criteria: 1, age less than 40 years; 2. Pregnancy; 3. History of radiographic contrast agent (barium) use in the past 7 days; 4. A weight greater than 450 lbs. A total of 10,175 subjects participated in this investigation. After excluding subjects with missing AAC scores (*n* = 7,035), incomplete physical activity data (*n* = 8), incomplete alcohol intake data (*n* = 1,244), incomplete BMI data (*n* = 12), incomplete cotinine (*n* = 48), and incomplete dietary data (*n* = 228), the final sample size used for the analysis was 1,600 (Figure 1).

### Assessment of the oxidation balance score

The OBS was calculated for a combination of 16 dietary elements and 4 lifestyle elements including five pro-oxidants and fifteen antioxidants, and all factors were screened by their prior information on their association with oxidative stress (14). The 16 dietary elements intake data points were obtained from the NHANES 2013–2014 Dietary Data Questionnaire and included fourteen antioxidants (dietary fiber, beta-carotene, riboflavin, niacin, total folate, vitamin B6, vitamin B12, vitamin C, vitamin E, calcium, magnesium, zinc, copper, and selenium) and two pro-oxidants (iron and total fat). Four sets of lifestyle data were obtained from the NHANES 2013–2014 questionnaires or examination data on the relevant topics and included three pro-oxidants (smoking, alcohol consumption, BMI) and one antioxidant (physical activity). Dietary data were collected through an automated multiple-pass method that provides an efficient and accurate way of collecting data for large-scale national surveys such as the NHANES (15). Two 24-h dietary reviews were conducted by a professional dietary investigator, the first face-to-face and the second completed by telephone 3 to 10 days later. Dietary data intake was calculated based on nutrient values from the USDA Dietary Research Food and Nutrition Database, and the average of the two reviews was taken as the result (16). Cotinine is the major metabolite of nicotine and can be a marker of both active smoking and exposure to or passive smoking in the tobacco environment (17); therefore, we used the serum cotinine concentration to assess tobacco exposure. Cotinine in serum specimens was detected by isotope dilution high-performance liquid measurement chromatography/atmospheric pressure chemical ionization tandem mass number spectrometry, and specimens were handled, stored, and shipped to the Laboratory Division, Science, the National Center for Environmental Health, and the Centers for Disease for analysis and were stored at-20°C prior to testing. Alcohol consumption was the mean value obtained by calculating the amount of any type of alcoholic beverage used by the participant on a daily basis over a 12-month period. BMI was calculated as weight (kg) divided by height (m) squared. For physical activity, we incorporated work-related high-intensity activities, moderate-intensity activities, and leisure-time activities, including walking or bicycling, high-intensity leisure activities, and moderate-intensity leisure activities. For the calculation of physical activity scores, we used the methodology of previous studies (18, 19); in short, physical activity score = metabolic equivalent (MET) score × frequency of each physical activity per week × duration of each physical activity. Vigorous Work Activity: MET = 8, Moderate Work Activity: MET = 4, Walking or Bicycling: MET = 4, Leisure Time Vigorous Physical Activity MET = 8, Leisure Time Moderate Physical Activity: MET = 4. The scores for all variables for each individual were summed to obtain their oxidative homeostasis score, with higher scores indicating greater exposure to antioxidants. Continuous variables were scored on a scale from 0 to 2 (for pro-oxidants from 0 to 2, and for antioxidants from 2 to 0) based on increments in tertiles. We categorized the OBS data into 4 groups according to quartiles: first quartile (Q1) (OBS = 5 ~ 14), second quartile (Q2) (OBS = 15 ~ 21), third quartile (OBS = 22 ~ 26), and fourth quartile (Q4) (OBS = 27 ~ 37) (Table 1).

**Table 1:** Components of the oxidation balance scores from the 2013–2014 NHANES database.

OBS components	Property	Male			Female		
		0	1	2	0	1	2
Dietary OBS components							
Dietary fiber(g/d)	A	<12.85	12.85–21.30	≥21.30	<11.90	11.90–18.00	≥18.00
β-Carotene (RE/d)	A	<685.00	685.00–2154.50.00	≥2154.50.00	<40.5	40.50–344.5	≥344.5
Riboflavin (mg/d)	A	<1.78	1.78–2.54	≥2.54	<1.46	1.46–2.05	≥2.05
Niacin (mg/d)	A	<22.47	19.31–31.37	≥31.37	<16.69	16.69–23.17	≥23.17
Total folate (mcg/d)	A	<315.00	315.00–468.00	≥468.00	<262.50	262.5–388.50	≥388.50
Vitamin B6 (mg/d)	A	<1.80	1.80–2.52	≥2.52	<1.38	1.38–1.97	≥1.97
Vitamin B12 (mcg/d)	A	<3.430	3.43–5.81	≥5.81	<2.47	2.47–4.29	≥4.29
Vitamin C (mg/d)	A	<40.00	40.0–95.35	≥95.35	<38.90	38.90–86.75	≥86.75
Vitamin E (ATE)(mg/d)	A	<6.65	6.17–10.31	≥10.31	<5.79	5.79–9.28	≥9.28
Calcium (mg/d)	A	<705.50	705.50–1054.00	≥1054.00	<615.50	615.50–931.50	≥931.50
Magnesium (mg/d)	A	<264.00	264–371.50	≥371.50	<219.5	219.50–303.50	≥303.50
Zinc (mg/d)	A	<9.295	9.295–13.40	≥13.40	<7.21	7.21–10.26	≥10.26
Copper (mg/d)	A	<1.01	1.01–1.43	≥1.43	<0.87	0.87–1.24	≥1.24
Selenium (mcg/d)	A	<103.80	103.80–146.10	≥146.10	<76.85	76.85–109.40	≥109.40
Total fat (g/d)	P	≥98.48	66.12–98.48	<66.12	≥78.93	54.32–78.93	<54.32
Iron (mg/d)	P	≥17.23	11.99–17.23	<11.99	≥13.77	9.67–13.77	<9.67
Lifestyle OBS components							
Physical activity (MET-minute/week)	A	<500	500–2,780	≥2,780	<280	180–1,600	≥1,600
Alcoholic drinks at past 12 months (drink/d)	P	≥4	2–4	<2	≥3	2–3	<2
Body mass index (kg/m2)	P	≥29.50	26.10–29.50	<26.10	≥31.00	25.20–31.00	<25.20
Cotinine(ng/mL)	P	≥0.191	0.02–0.191	<0.02	≥0.067	0.020–0.067	<0.02

A, antioxidant; P, pro-oxidant; RE, retinal equivalent; ATE, alpha-tocopherol equivalent; MET, metabolic equivalent.

### Assessment of abdominal aortic calcification

**Figure 2:**
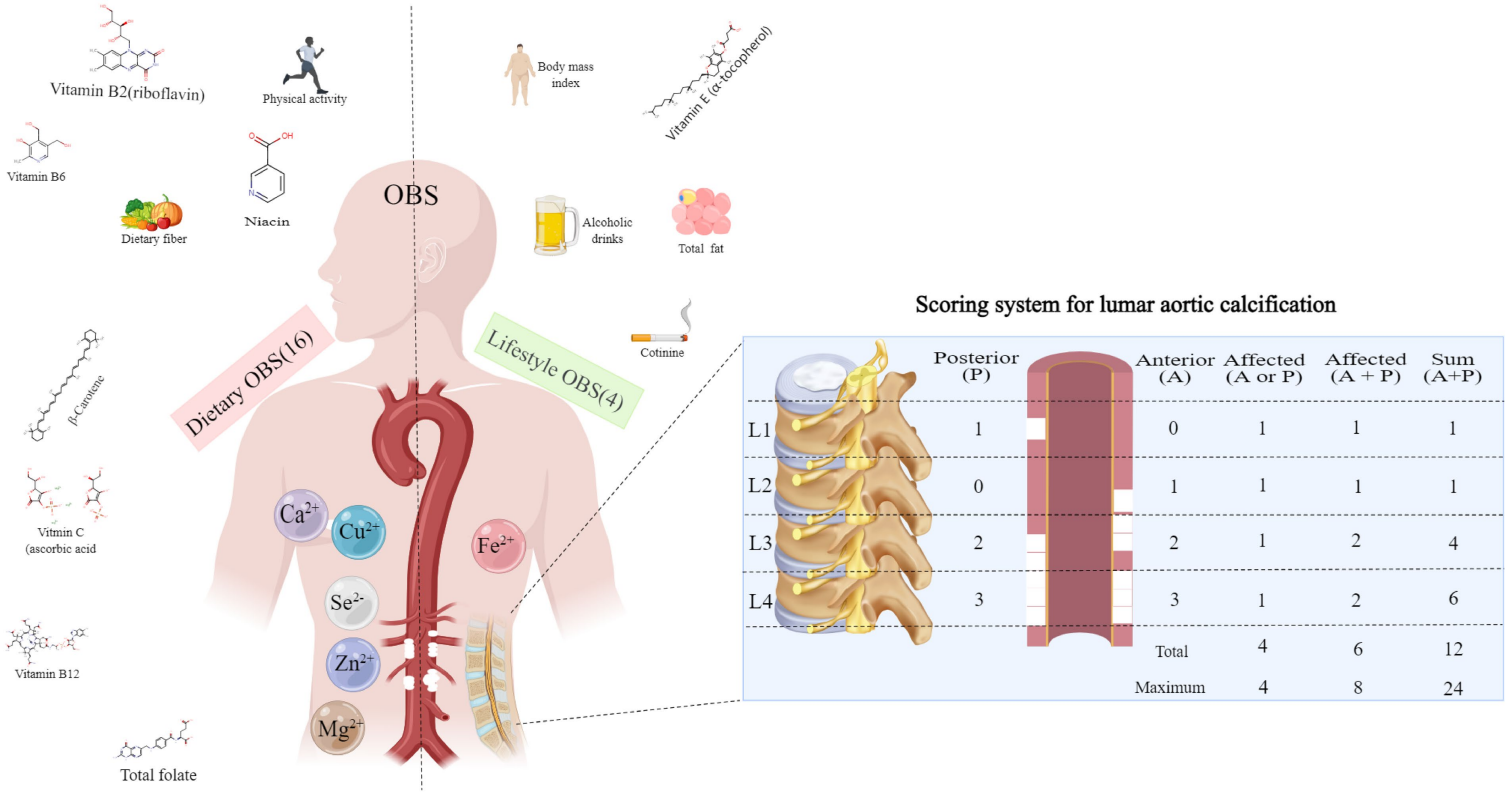
Oxidative balance score component and abdominal aortic calcification score criteria. OBS, oxidation balance score.

The AAC score was derived from NHANES 2013–2014 examination data. NHANES sent trained evaluators to evaluate lateral lumbar spine images scanned by dual-energy X-ray absorptiometry using the widely recognized Kauppila scoring method to assess the severity of calcified blood vessels (20). The AAC score is used to assess the severity of AAC, with higher scores indicating a more pronounced degree of calcification. The Kauppila scoring system divides the abdominal aortic wall into 4 contiguous segments that directly correspond to the L1 to L4 vertebral regions. The Kauppila scoring system divides the abdominal aortic wall into 4 contiguous segments that correspond directly to the L1 to L4 vertebral regions. Each segment is given a scores (0–6) based on the degree of calcium deposition, and the sum of the scores for all segments yields the final AAC scores (0–24). The specifics of the OBS and AAC scores for humans are shown in Figure 2.

### Assessment of covariates

The questionnaires in this study were administered by trained interviewers using a computer-assisted personal interviewing system in the home. Demographic data were collected using a standardized survey in which race and ethnicity were divided into five groups: Mexican American, non-Hispanic white, non-Hispanic black, other Hispanic, and other race/multiracial. Education was categorized into two groups: high school and below and college and above. Poverty was defined as having a ratio of family income to poverty (the ratio of family income to poverty) ≤ 1.3 (21). Triglycerides, cholesterol, uric acid, and creatinine were obtained from standardized biochemical tests. DM was defined as the use of glucose-lowering medications or a diagnosis of DM, a glycohemoglobin level ≥ 6.5%, and a fasting blood glucose ≥126 mg/dL or a 2-h blood glucose ≥200 mg/dL (22). Hypertension was defined as the use of antihypertensive medications, a diagnosis of hypertension, or three consecutive systolic blood pressure readings ≥140 mmHg or diastolic blood pressure ≥ 90 mmHg (23). Postmenopausal women were defined as menopausal women who had their uterus removed or both ovaries removed or who were 55 years of age or older (24).

### Statistical analysis

All analyses were performed with appropriate sampling weights and using R statistical software (Version 4.2.2, http://www.R-project.org, The R Foundation), the Statistical Package for Social Science (SPSS) (version 28, https://www.malavida.com/en/soft/ibm-spss-statistics/), and the Free Statistical analysis platform (Version 1.9, Beijing, China) to conduct a complex multistage cluster investigation study.^2^ Regression in SPSS software was used to fill in missing data. This study presents a secondary analysis of publicly available data from the NHANES dataset. To ensure unbiased estimates and accurate significance levels, it is recommended to use sampling weights and sampling design variables for all analyses. Therefore, we followed the NHANES analytical guidelines and included complex sampling designs and sampling weights in our analyses. Our study data were obtained from household interviews and dietary data collected during the NHANES survey. We used the WTDR2D weights for analysis following the NHANES recommendations for survey sample weighting analysis guidelines.

Continuous variables at baseline are expressed as the weighted mean (SD), and categorical variables are expressed as weighted percentages. Groups Q1, Q2, Q3, and Q4 were categorized by quartiles according to the OBS. For categorical continuous variables and non-normal continuous variables, the Rao–Scott chi-square test and the Kruskal–Wallis test were used to test for differences in the characteristics of the variables across OBS groups (quartiles), respectively. In contrast, for normally distributed continuous variables, one-way analysis of variance (ANOVA) was used to analyze the differences between quartile groups. Weighted linear regression was used to explore the correlation between AAC and OBS. To further explore the association between OBS and AAC in different populations, subgroup analyses based on sex, age, BMI, race, education and poverty status were conducted, and trend tests were performed. Three models were constructed. The crude model was not adjusted for any covariates. Model 1 was adjusted for sex, age, and BMI. Model 2 was modeled by adding race, education and poverty to Model 1. Stratified smoothed curve fitting was used to explore the potential relationship between AAC and OBS between men and women and to further explore the potential relationship before and after menopause among women. Statistical tests were two-sided, and *p* values <0.05 indicated statistical significance.

## Results

### Baseline characterization

The study included 1,600 middle-aged and elderly participants. The missing data were as follows: household income to poverty ratio (6.44%, 103/1,600), uric acid (0.50%, 8/1,600), triglycerides (0.50%, 8/1,600), cholesterol (0.50%, 8/1,600), creatinine (0.38%, 6/1,600), and total calcium ions (1.00%, 16/1,600). The missing data were filled in using regression in SPSS software. The population was composed of 49.78% males and 50.22% females. Only 13.26% of the population had diabetes, and 46.26% had hypertension. The OBS quartile groups included 412, 453, 370, and 365 patients, respectively. The first quartile group had 51.39% males and 48.61% females, with a mean age of 56.29 (11.13) years. The second quartile group of OBS comprised 53.64% males and 46.36% females, with a mean age of 55.74 (10.37) years. The third quartile group of OBS comprised 48.43% males and 51.57% females, with a mean age of 57.79 (10.69) years. The fourth quartile group consisted of 45.45% males and 54.55% females, with a mean age of 56.41 (11.38) years.

Table 2 shows a comparison of the baseline data, AAC, and other indices among the 4 patient groups. There were no significant differences in sex or age among the 4 groups (all *p* > 0.05). However, BMI gradually decreased among the 4 groups (*p* < 0.001). Regarding race and education level, the percentage of non-Hispanic whites increased progressively from the first quartile to the fourth quartile group of OBS (*p* < 0.001). Similarly, there was a progressively greater percentage of individuals with college-level literacy or above in all 4 groups (*p* < 0.001). Additionally, in terms of the household income-to-poverty ratio, those with a poverty level (≤1.3) represented the lowest percentage in the third quartile group, while those without a poverty level (>1.3) represented the lowest percentage in the first quartile group (*p* < 0.001). The proportions of individuals with DM or hypertension gradually decreased from the first to the fourth quarter (*p* < 0.001).

**Table 2:** Baseline characteristics by oxidation balance score quartile.

Variables	Overall (*N* = 1,600)	Q1 (*N* = 412)	Q2 (*N* = 453)	Q3 (*N* = 370)	Q4 (*N* = 365)	*P*-valve
Sex (%)						0.473
Male	49.78	51.39	53.64	48.43	45.45	
Female	50.22	48.61	46.36	51.57	54.55	
Age (mean (SD))	56.53 (10.90)	56.29 (11.13)	55.74 (10.37)	57.79 (10.69)	56.41 (11.38)	0.268
BMI(kg/m^2^) (mean (SD))	28.14 (5.44)	30.08 (5.69)	28.72 (5.32)	27.64 (5.00)	26.27 (5.06)	<0.001
Race (%)						0.006
Mexican American	5.98	6.45	6.69	4.34	6.33	
Other Hispanic	4.1	3.94	5.2	3.23	3.86	
Non-Hispanic White	74.13	67.17	72.62	77.84	78.43	
Non-Hispanic Black	9.01	15.46	9.39	6.73	5.04	
Other Race	6.78	6.98	6.09	7.85	6.35	
Education (%)						<0.001
High school graduate or less	28.71	40.39	29.88	21.11	24.27	
College or more	71.29	59.61	70.12	78.89	75.73	
Poverty (%)						<0.001
≤1.3	13.75	23.21	13.64	9.17	9.80	
>1.3	86.25	23.22	86.36	90.83	90.2	
Diabetes mellitus (%)						0.025
No	86.74	81.13	85.90	88.53	90.92	
Yes	13.26	18.87	14.10	11.47	9.08	
Hypertension (%)						<0.001
No	53.74	44.09	49.52	50.50	70.00	
Yes	46.26	55.91	50.48	49.50	30.00	
Glycohemoglobin	5.5 [5.3, 5.8]	23.23	5.50 [5.30, 5.80]	5.5 [5.2, 5.8]	5.4 [5.2, 5.7]	0.014
AAC score (median [range])	0.00 [0.00, 22.00]	0.00 [0.00, 21.00]	0.00 [0.00, 21.00]	0.00 [0.00, 17.00]	0.00 [0.00, 22.00]	0.015
Cholesterol (mmol/L) (mean (SD))	5.15 (1.08)	5.28 (1.24)	5.07 (1.08)	5.01 (1.00)	5.25 (0.99)	0.038
Creatinine (umol/L) (median [IQR])	79.56 [66.30, 91.94]	79.56 [66.30, 95.47]	80.44 [66.87, 92.82]	77.79 [67.18, 89.28]	77.79 [65.42, 87.52]	0.410
Triglycerides, (mmol/L) (median [IQR])	1.43 [0.97, 2.27]	1.79 [1.14, 2.43]	1.48 [1.03, 2.34]	1.28 [0.92, 1.94]	1.28 [0.87, 2.08]	<0.001
Uric acid (umol/L) (mean (SD))	324.18 (81.59)	348.47 (85.85)	330.99 (81.88)	313.13 (74.54)	305.68 (77.63)	<0.001
Total calcium (mmol/L) (mean (SD))	2.37 (0.09)	2.37 (0.09)	2.36 (0.08)	2.38 (0.11)	2.37 (0.08)	0.286

BMI, body mass index; AAC, abdominal aortic calcification; Q1 ~ Q4: the first to fourth quantiles of the oxidation balance score.

The blood test results indicated that there was no significant difference in cholesterol or creatinine levels among the 4 groups (all *p* > 0.05). However, triglyceride and uric acid levels decreased gradually from the first to the fourth quartile of OBS, and the difference was statistically significant (all *p* < 0.05). Representing the AAC scores as medians and interquartile ranges (IQRs) was challenging due to the non-normal distribution across the four groups, with the majority of scores being smaller than 1. Therefore, we presented the median (minimum, maximum) AAC score for each group. The results showed statistically significant differences in AAC scores among the 4 groups (*p* < 0.001).

### Associations between OBS quartiles and AAC

**Figure 3:**
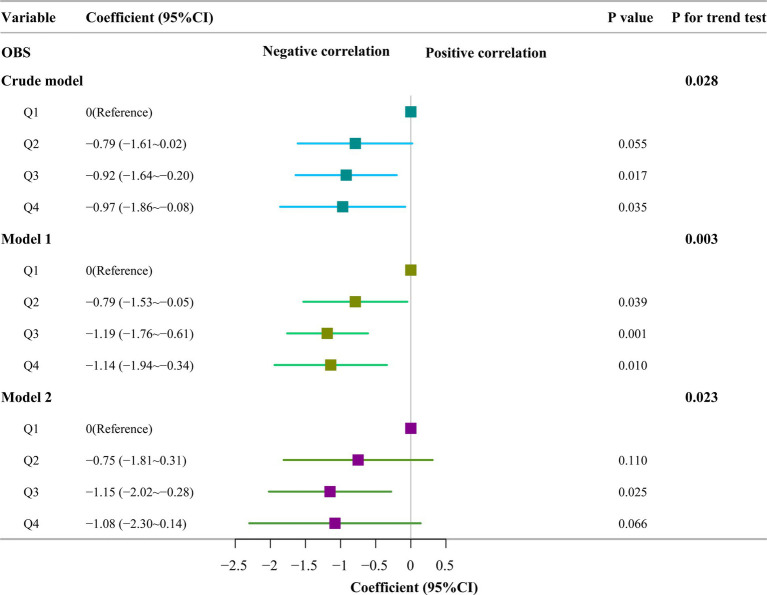
Multivariate adjusted linear regression for oxidation balance score quartile and abdominal aortic calcification score. Crude model: no covariates were adjusted; Model 1: adjusted for sex, age and body mass index; Model 2: adjusted for variables in Model 1 plus race, education and poverty; OBS, oxidation balance score; Q1 ~ Q4: the first to fourth quantiles of the oxidation balance score.

A diet-weighted (WTDR2D) linear regression model was used to explore the relationships between OBS quartiles and AAC. Weights were based on census data, ensuring our findings’ generalizability to the broader US population. In the crude model analysis, no adjustment was made for any covariates. AAC scores were lower in the third and fourth quartiles than in the first quartile group (Q3: coefficients [coef], −0.92 [95% CI, −1.64 ~ −0.20], *p* = 0.017; Q4: coefficients [coef], −0.97 [95% CI, −1.86 ~ −0.08; *p* = 0.035]), whereas AAC scores were similar in the second quartile group of OBS (Q2: coefficients [coef], −0.79 [95% CI, −1.61 ~ 0.02], *p* = 0.055). Model 1 (Model 1 is adjusted for sex, age, and BMI.), the AAC scores were sequentially lower in the second, third, and fourth quartile groups than in the first quartile group (Q2: coefficients [coef], −0.79 [95% CI, −1.53 ~ −0.05], *p* = 0.039; Q3. coefficients [coef], −1.19 [95% CI, −1.76 ~ −0.61], *p* = 0.001; Q4: coefficients [coef], −1.14 [95% CI, −1.94 ~ −0.34]; *p* = 0.010); Model 2 (Model 2 was modeled by adding race, education and poverty to Model 1.), the AAC scores were sequentially lower in the third quartile group of OBS than in the first quartile group (Q3: coefficients [coef], −1.15 [95% CI, −2.02 ~ −0.28], *p* = 0.025), and the AAC scores were similar to those in the second (Q2: coefficients [coef], −0.75 [95% CI, −1.81 ~ 0.31]; *p* = 0.110) and fourth (Q4: coefficients [coef], −1.08 [95% CI, −2.30 ~ 0.14]; *p* = 0.066) quartile groups of OBS and were significantly different for the trend test in all three models (all *p* < 0.05) (Figure 3).

### Relationship between dietary and lifestyle OBS quartiles and AAC

**Figure 4:**
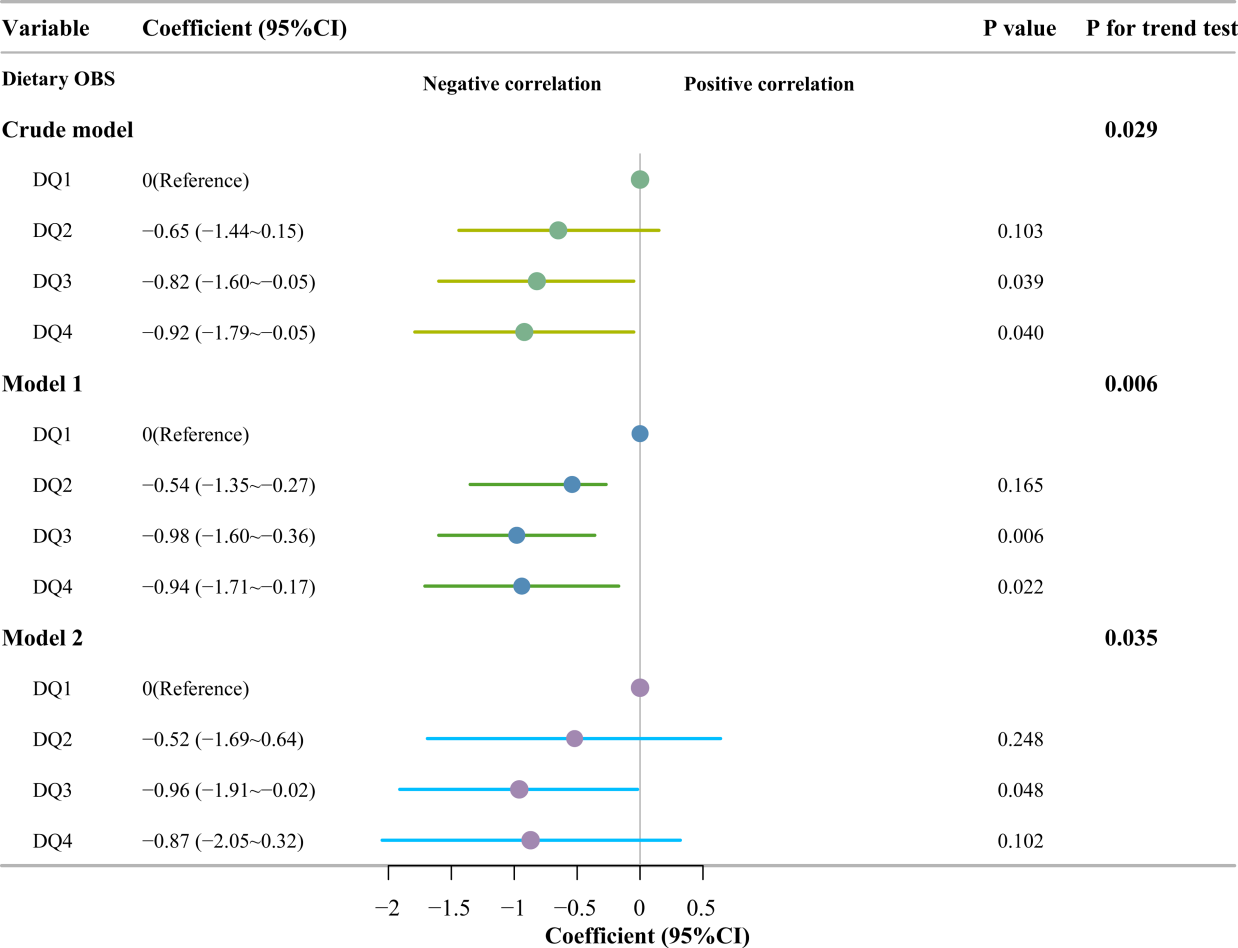
Multivariate linear regression of dietary OBS quartile and abdominal aortic calcification scores. Crude model: no covariates were adjusted; Model 1: Adjusted for sex, age and body mass index; Model 2: Adjusted for variables in Model 1 plus race, education and poverty; OBS, oxidation balance score; DQ1 ~ DQ4: The first to fourth quantiles of dietary oxidation balance score.

This study subdivided OBS into dietary OBS and lifestyle OBS, which are derived from a combination of 16 dietary elements and 4 lifestyle elements, and conducted a weighted linear regression of their respective quartiles against the AAC. In one of the weighted linear regressions of dietary OBS with AAC, the results showed that In the crude model analysis, no adjustment was made for any covariates. AAC scores were sequentially lower in the third and fourth quartile groups compared with those in the first quartile group (Q3: coefficients [coef], −0.92 [95% CI, −1.64 ~ −0.20], *p* = 0.017; Q4: coefficients [coef], −0.97 [95% CI, −1.86 ~ −0.08]; *p* = 0.035), and the second quartile group had the similar AAC scores with the first quartiles(Q2: coefficients [coef], −0.79 [95% CI, −1.61 ~ 0.22], *p* = 0.055); In Model 1 (Model 1 is adjusted for sex, age, and BMI), AAC scores were sequentially lower in the second, third and fourth quartile groups of dietary OBS compared with those in the first quartile group of dietary OBS (Q2: coefficients [coef], −0.79 [95% CI, −1.53 ~ −0.05], *p* = 0.039; Q3: coefficients [coef], −1.19 [95% CI, −1.76 ~ −0.61], *p* = 0.001; Q4: coefficients [coef], −1.14 [95%CI, −1.94 ~ −0.34]; *p* = 0.010); In Model 2 (Model 2 was modeled by adding race, education and poverty to model 1.), the AAC scores were lower in the third quartile group of dietary OBS than in the first quartile group of dietary OBS (Q3: coefficients [coef], −0.96 [95% CI, −1.96 ~ −0.02], *p* = 0.048), and the AAC scores of the second and fourth quartile groups of OBS were similar to those of the first quartile (Q2: coefficients [coef], −0.52 [95% CI, −1.69 ~ 0.64], *p* = 0.248; Q4: coefficients [coef], −0.87 [95% CI, −2.05 ~ 0.32], *p* = 0.102). The trend tests of the three models revealed statistically significant differences (all *p* < 0.05) (Figure 4).

**Figure 5:**
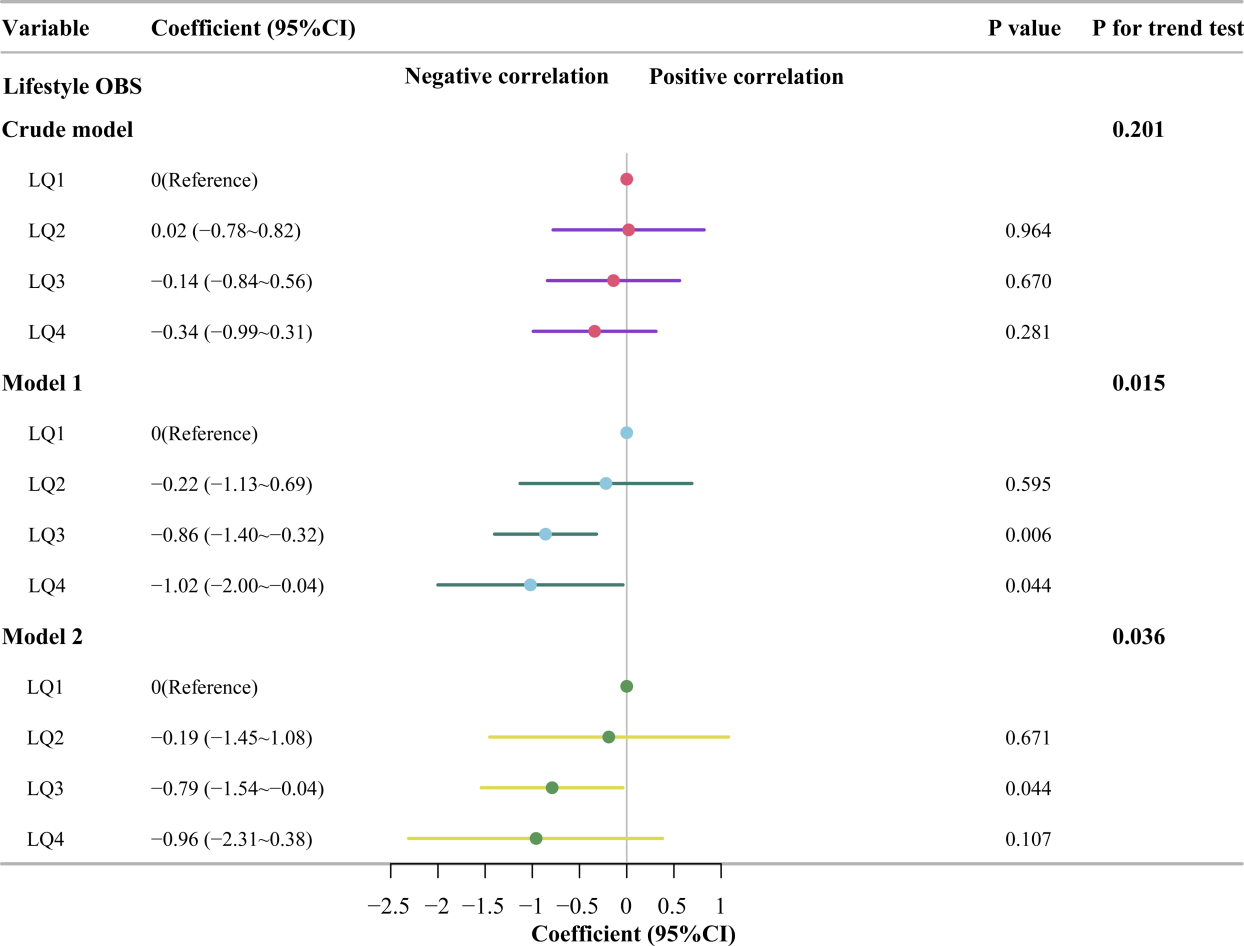
Multivariate linear regression of lifestyle OBS quartiles and abdominal aortic calcification scores. Crude model: no covariates were adjusted; Model 1: Adjusted for sex, age and body mass index; Model 2: Adjusted for variables in Model 1 plus race, education and poverty; OBS, oxidation balance score; LQ1 ~ LQ4: The first to fourth quantiles of lifestyle oxidation balance score.

In analyzing the association between lifestyle OBS and AAC, the results showed that in the crude model, the AAC scores for the second, third, and fourth quartiles of lifestyle OBS were similar to those of the first quartile (Q2: coefficients [coef], −0.02 [95% CI, −0.78 ~ 0.82], *p* = 0.964; Q3: coefficients [coef], −0.14 [95% CI, −0.84 ~ 0.56], *p* = 0.670; Q4: coefficients [coef], −0.34 [95% CI, −0.99 ~ 0.31], *p* = 0.281). Model 1 (Model 1 is adjusted for sex, age, and BMI.), the third and fourth quartiles of lifestyle OBS had lower AAC scores than did the first quartile group of lifestyle OBS (Q3: coefficients [coef], −0.86 [95% CI, −1.40 ~ −0.32], *p* = 0.006; Q4: coefficients [coef], −1.02 [95% CI, −2.00 ~ −0.04], *p* = 0.044), while the second quartile group of lifestyle OBS had similar AAC scores to those of the first quartile (Q2: coefficients [coef], −0.22 [95% CI, −1.13 ~ 0.69]; *p* = 0.595). Model 2 (Model 2 was modeled by adding race, education and poverty to Model 1.), the AAC scores were lower in the third quartile group of lifestyle OBS than in the first quartile group (Q3: coefficients [coef], −0.79 [95% CI, −1.54 ~ −0.04], p = 0.044), and the AAC scores of the second and fourth quartile groups were similar to those of the first quartile group (Q2: coefficients [coef], −0.19 [95% CI, −1.45 ~ 1.08], *p* = 0.671; Q4: coefficients [coef], −0.96 [95% CI, −2.31 ~ 0.38], *p* = 0.107). The trend tests of the three models revealed statistically significant differences (*p* < 0.05) (Figure 5).

### Subgroup analysis of OBS quartiles associated with AAC in the total population

**Figure 6:**
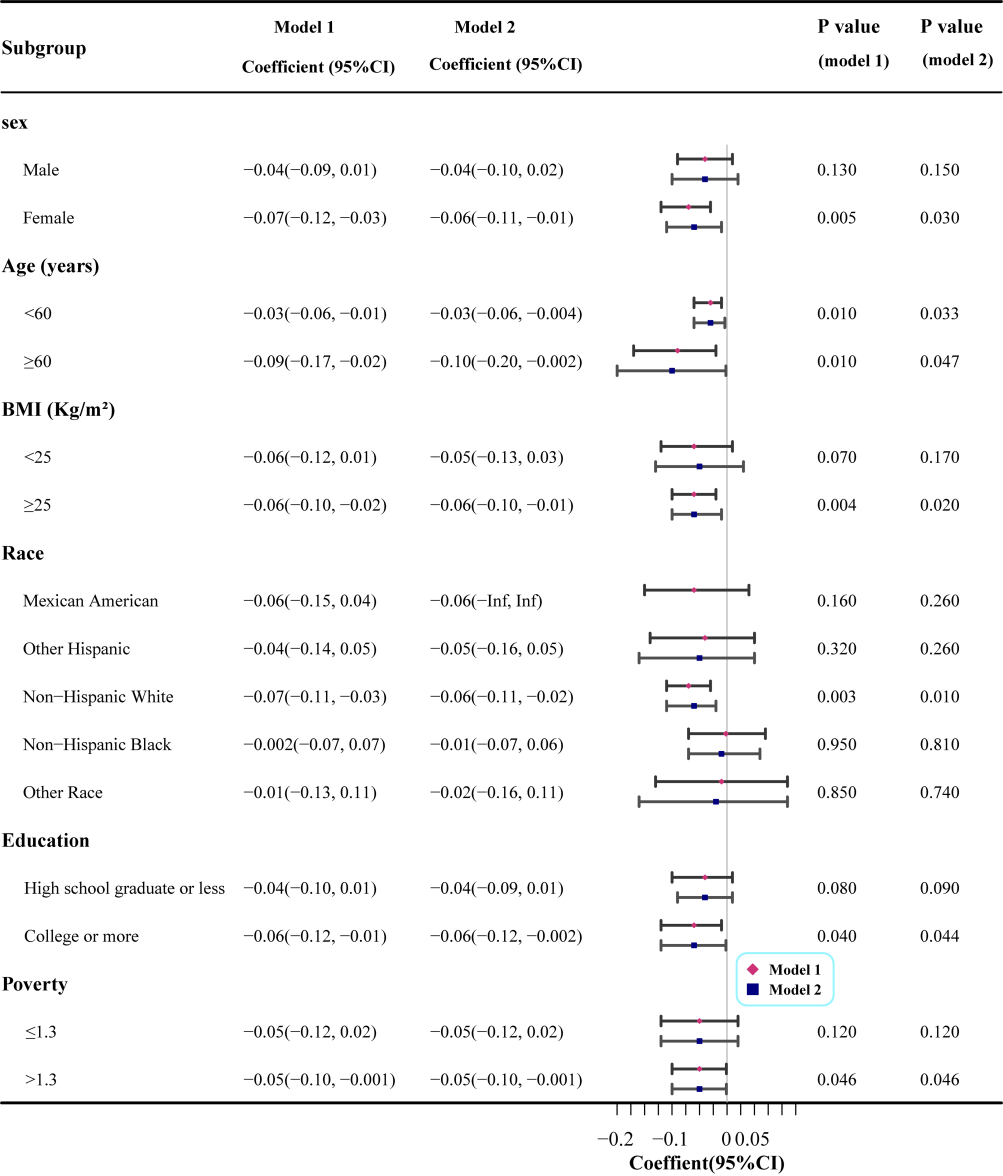
Subgroup analysis of linear regression between oxidation balance score quartile and abdominal aortic calcification score. Model 1: Adjusted for sex, age and body mass index; Model 2: Adjusted for variables in Model 1 plus race, education and poverty.

To investigate the potential association between OBS and AAC, we conducted subgroup analyses of the covariates using weighted linear regression. The detailed results are shown in Figure 6. Two analytical models were used in this study. Model 1 was adjusted for sex, age, and BMI, and Model 2 further included variables such as race, education and poverty based on Model 1 to comprehensively assess the effects of factors on the association between OBS and AAC scores. The analysis results indicate that there were no significant differences in the association between OBS and AAC scores between males in the sex subgroup in either Model 1 or Model 2 (both *p* > 0.05), in contrast to females who showed statistically significant associations between OBS and AAC scores in both models (both *p* < 0.05). In the age subgroup analyses, the association between OBS and AAC scores was statistically significant (all *p* < 0.05) regardless of age (using 60 years as the cutoff), suggesting that age did not significantly affect the relationship. Upon further analysis of the race subgroup data, we found that only Mexican Americans demonstrated a significant correlation between OBS and AAC scores in both Model 1 and Model 2 (*p* < 0.05). In contrast, the other races did not show a significant correlation between OBS and AAC scores in either model (all *p* < 0.05). In the subgroup with a literacy level of high school graduate or less, there was no clear correlation between the association of OBS and AAC scores in either Model 1 or Model 2 (both *p* > 0.05). Conversely, for those with a college education or above, there were significant correlations (all *p* < 0.05). In the subgroup analyses of family income-to-poverty ratios, it was found that OBS and AAC scores did not exhibit a significant correlation in either model among those below the poverty line (poverty ≤1.3); however, among those not in poverty (poverty >1.3), the correlation between OBS and AAC scores was found to be statistically significant (both *p* < 0.05) in both models 1 and 2.

### Analysis of the association between OBS and AAC in males versus females or premenopausal versus postmenopausal females

**Figure 7:**
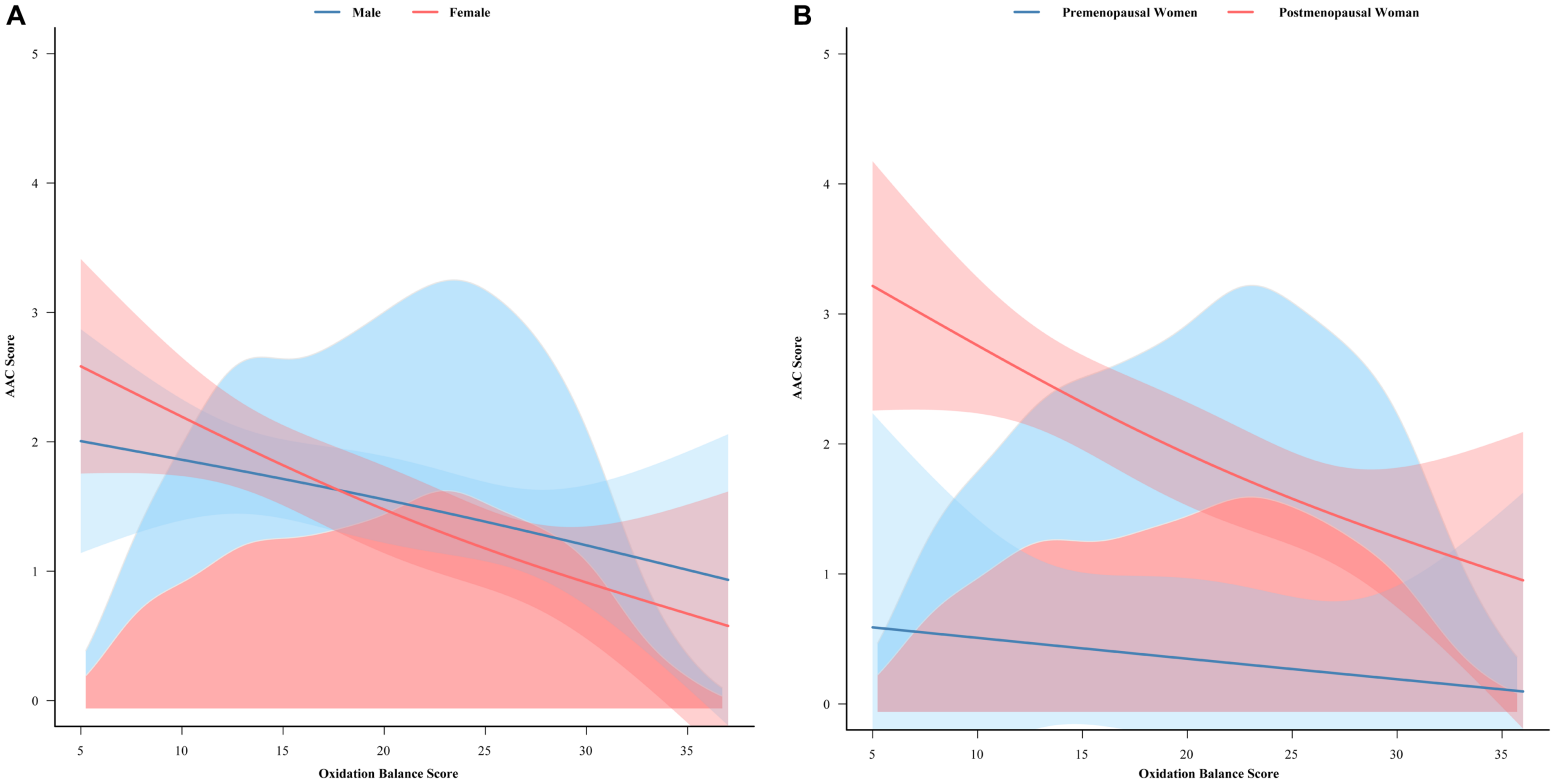
Smooth curve fitting analysis of the association between the oxidative balance score and the AAC score. **(A)** Shows the smooth curve fitting analysis of the correlation between the oxidation balance scores and the AAC scores of male and female. **(B)** Shows the smooth curve fitting analysis of the correlation between the oxidative balance score and the AAC score of premenopausal and postmenopausal women. AAC, abdominal aortic calcification.

Smoothed curve fitting was performed based on the results of subgroup analyses that revealed a difference between males and females in the association between OBS and AAC scores. The analysis showed that as OBS increases, females are more likely to have lower AAC scores than males are (Figure 7A). We further investigated the association between OBS and AAC scores among females and males using weighted linear regression. The findings revealed a significant negative correlation between OBS and AAC scores specifically among females, whereas no such association was identified among males (Supplementary Table S1). A dramatic decrease in estrogen levels around menopause in women may have an effect on calcification. Smoothed curve fitting was performed separately for premenopausal and postmenopausal women. The results showed that in postmenopausal women, the AAC scores decreased significantly with increasing OBS. However, in premenopausal women, the decreases were also significant but less pronounced than those in postmenopausal women (Figure 7B). We further explored the association between OBS and AAC scores among premenopausal and postmenopausal women through the application of weighted linear regression. Our findings demonstrated a statistically significant negative correlation between OBS and AAC scores exclusively in postmenopausal women, while no such association was evident in premenopausal women (Supplementary Table S2).

### Analysis of potential mediators of the relationship between OBS and AAC scores

**Figure 8:**
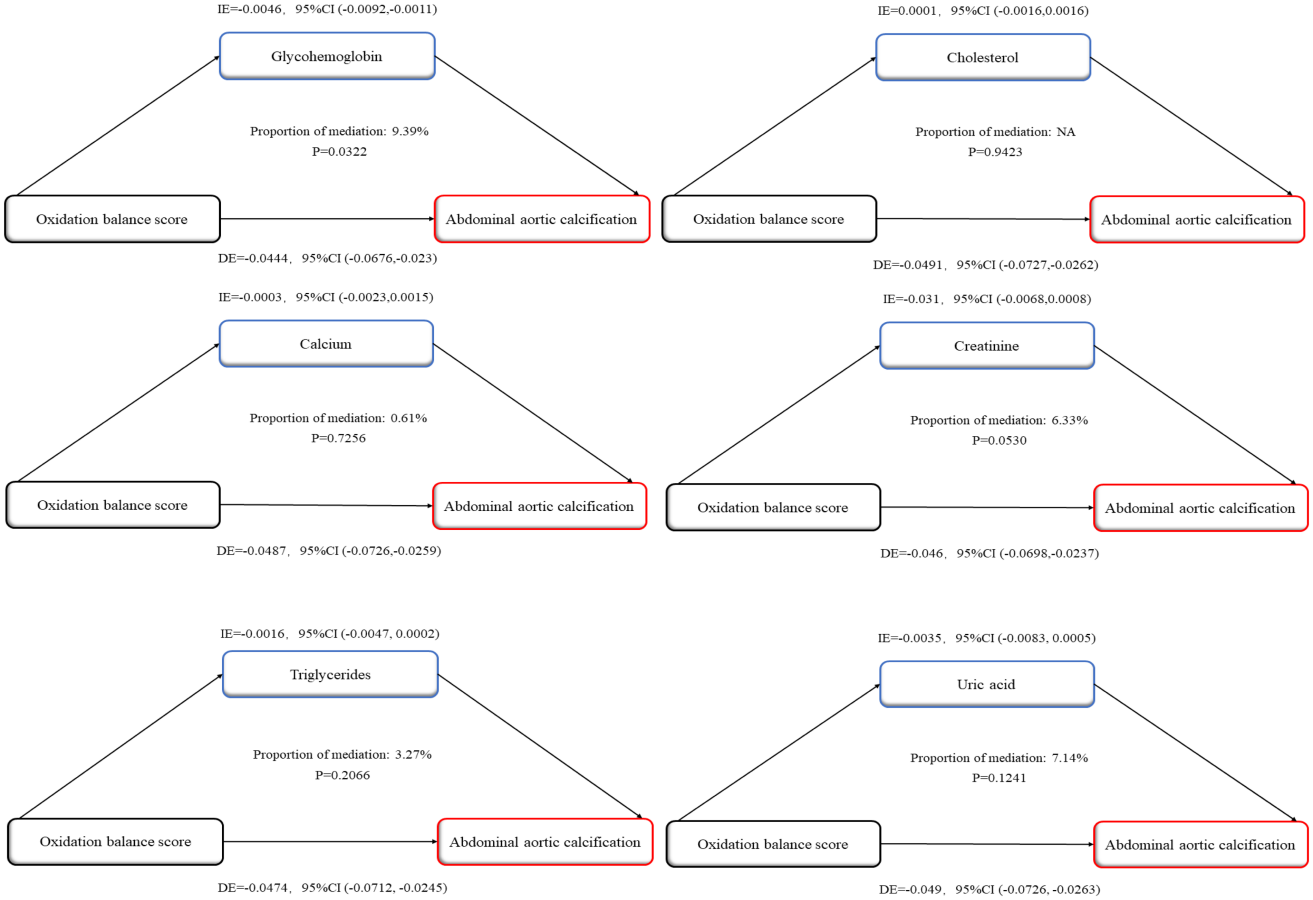
Mediation analysis of oxidation balance score and abdominal aortic calcification score. IE, indirect effect; DE, direct effect; Proportion of mediation = IE/(DE + IE).

This study evaluated whether glycohemoglobin, triglyceride, cholesterol, creatinine, uric acid, and calcium ion levels mediated the negative association between OBS and AAC scores. Figure 8 shows that glycohemoglobin mediated this association by 9.39% (*p* = 0.0322), while triglycerides, cholesterol, creatinine, uric acid, and calcium ions did not significantly mediate this association. Furthermore, OBS was linked to a reduction in glycohemoglobin levels, and glycohemoglobin was negatively correlated with AAC scores (Supplemental Table S3), which is consistent with the findings of a “statistically significant association between X and M” and a “statistically significant association between M and Y,” indicating the occurrence of intermediate effects.

## Discussion

This study is based on a national survey in which a significant negative association between OBS and AAC was found in the overall population. After adjusting for covariates, including sex, age, race, education level, the ratio of family income to poverty, DM status, and hypertension status, the AAC scores decreased as the number of OBS quartiles increased. Moreover, both dietary OBS and lifestyle OBS were inversely related to AAC scores, and dietary OBS was more significantly related to AAC scores. Finally, subgroup analyses showed that we found women to be more sensitive than men in the above relationships and that postmenopausal women were also significantly more sensitive than premenopausal women in both inverse relationships. Our findings suggest the importance of anti−/pro-oxidant dietary elements and lifestyles targeting the enhancement of OBS for the reduction of AAC, especially for postmenopausal women.

OBS is a comprehensive indicator of the human body through the combination of 16 anti−/pro-oxidant dietary elements and 4 anti−/pro-oxidant lifestyles. Each unit increase in the quartiles of OBS in the overall population corresponded to a lower AAC score. Our findings are similar to those of previous clinical studies confirming the benefits of dietary interventions. For example, Jia et al. (25) reported that Higher dietary vitamin C intake is associated with lower AAC. In animal experiments, the antioxidant Tempol was also found to reduce systemic oxidative stress levels and inhibit vascular smooth muscle cell osteogenic differentiation and arterial calcification in uremic rats (26). In addition, a single component of the diet has been found to influence the occurrence of AAC in some studies. Chen et al. (27) reported that higher dietary zinc intake was independently correlated with a lower chance of suffering from severe AAC.

Different lifestyles may also have an impact on AAC scores, such as smoking, alcohol consumption and exercise, all of which are included as components of the OBS. The results of our study suggest that AAC scores will be lower in the fourth quartile of lifestyle OBS than in the first quartile of lifestyle OBS. This finding suggests that a higher lifestyle OBS may similarly be associated with a lower AAC scores. Our findings are similar to those of previous studies. A recent Mendelian randomization study revealed a causal association between alcohol consumption and vascular calcification (28). A study involving 2,369 participants revealed a significant correlation between nicotine in cigarettes and AAC (29). Another study revealed that moderate to vigorous exercise had a beneficial effect on AAC (30). Body weight is an important indicator of cardiovascular health, and BMI-defined obesity has been found to be closely associated with AAC in US adults (31). However, a single ingredient of OBS may not be sufficient to explain its anti−/pro-oxidant effects in humans. Our study differs from others in that the OBS in our study is a comprehensive index that combines most of the oxidative/antioxidative substances involved in the human body, which provides a more accurate judgment of the antioxidant status of the human body compared to a single component. Although our results similarly confirm the beneficial effects of antioxidants on vascular calcification, there are studies that do not find such phenomena. In a double-blinded, randomized, placebo-controlled study, sodium thiosulfate, which has antioxidant properties, was found to have a positive effect on the progression of calcification in the iliac arteries and heart valves, among others, but no significant effect was observed in the AAC group (32). The reason may be that our study was based on multistage sampling with a wider and larger population, which will be more reliable.

Our study also revealed that females were more sensitive to OBS than males were, indicating that for every 1-point change in OBS, a more pronounced trend of change in AAC was observed in females. This suggests that we hypothesize that healthy females may be more inclined to consume a high-OBS diet/lifestyle. This may be because males may have more unhealthy habits, such as smoking or drinking alcohol, than females, resulting in a greater prevalence of the disease (33). Moreover, there are differences in sex hormone levels between males and females. A study of 1,287 participants revealed an inverse relationship between testosterone levels and coronary artery calcification in older men (34). Animal studies have also shown that androgens promote atherosclerosis and cardiovascular damage in female mice and are associated with decreased estrogen receptor expression (35). Considering the different levels of sex hormones in women pre-and postmenopause, we again performed a smooth curve-fitting correlation analysis between OBS and AAC in women pre-and postmenopause. The results showed that AAC in postmenopausal women was significantly more affected by OBS, meaning that for every 1 point change in OBS, the trend of change in AAC in postmenopausal women was more pronounced. A study of 9,374 postmenopausal female participants revealed that early menopause was associated with increased cardiovascular risk in postmenopausal women after more than 10 years of follow-up (36). Another study involving 144,260 postmenopausal women revealed an association between premature natural menopause and surgical menopause and cardiovascular disease after a median of 7 years of follow-up (37). This suggests that postmenopausal women are more likely to be at risk of vascular disease. Our study further suggests that higher OBS may be associated with lower risk of vascular calcification in postmenopausal women, but a larger prospective study is needed to validate this finding. Furthermore, the present study revealed that glycohemoglobin partially mediated the negative correlation between OBS and AAC scores, which is consistent with previous research. It is important to note that all the evaluations presented in this study are objective and supported by previous studies. For instance, an observational study of 1799 adults revealed a negative association between glycohemoglobin and AAC (38). Hyperglycemia causes lipid peroxidation damage, vascular smooth muscle cell apoptosis, and the induction of osteo lipoprotein expression (39, 40). A reduction in OS and optimal antioxidant intake are potentially beneficial factors for controlling the risk of diabetes (41). Therefore, our findings are robust and reliable.

## Strengths and limitations

Although some elements have been found to be associated with AAC, our study builds on this finding by using most of the dietary and lifestyle anti/promotive oxidative stressors (i.e., OBS) that cover a large part of the population’s daily life, which is a much more comprehensive and integrated indicator that describes the population’s daily life. Thus, the clinical significance of our study lies in the discovery of a negative correlation between OBS and AAC scores within the general population, which provides a basis for the development of targeted dietary and lifestyle interventions. In addition, separate subgroup analyses were conducted to investigate the potential association between OBS and AAC. The results showed that there was a more significant trend in the association between OBS and AAC in women than in men. Furthermore, smoothed curve fitting confirmed that postmenopausal women tended to have a more significant negative correlation between the two variables than premenopausal women did. The clinical significance of this study lies in determining whether the lower AAC scores observed in postmenopausal women may be associated with their daily food intake or lifestyles that contribute to higher OBS, while also suggesting that physicians and health professionals may need to consider gender and menopausal status when assessing and managing AAC risk. Finally, the present study revealed that glycohemoglobin partially mediated the negative correlation between OBS and AAC scores. Therefore, this study posits that glycohemoglobin may be associated with both of these themes, and effective control of glycohemoglobin could also aid in reducing the occurrence of AAC, which may hold particular significance for the clinical management of patients with DM.

This study has several limitations. First, the diet/lifestyle data were collected using a questionnaire format based on the NHANES design, which may have resulted in less accurate recall. However, we included a wide range of people and a population sample with multistage sampling to compensate for this deficiency. Second, as our study was cross-sectional, we cannot draw any conclusions about causality. Third, the NHANES used a complex sampling method, but it did not include the hospitalized population in the study. This may raise concerns about the incomplete assessment of critically ill patients.

## Conclusion

This study analyzed a nationally representative group of U.S. adults and revealed a potential negative association between OBS and AAC. The study also revealed that women were more sensitive to this association than men were, and postmenopausal women showed a more significant trend than premenopausal women did. Additionally, glycohemoglobin may have played a partial mediating role in the negative association between OBS and AAC.

## Data Availability

The original contributions presented in the study are included in the article/Supplementary material, further inquiries can be directed to the corresponding authors.

## Footnotes

^1^ https://www.cdc.gov/nchs/nhanes/index.htm

^2^ https://www.r-project.org/
