# Supplementary material for: Associations between oxidation balance score and abdominal aortic calcification, and the mediating role of glycohemoglobin: a nationally representative cross-sectional study from NHANES
Source: Front Nutr. 2025 Jan 20;12:1469449. doi: 10.3389/fnut.2025.1469449 (PMC11792858; doi:10.3389/fnut.2025.1469449)
Supplement: Supplementary file 1 [file Table_1.docx]

**Supplemental Table S1. Weighted linear regression between AAC score and OBS quartile in male or female**

| Variables | OBS quartile, [Coefficient (95%CI), P] | | | | P-trend |
| --- | --- | --- | --- | --- | --- |
|  | Q1 | Q2 | Q3 | Q4 |  |
| male | | | | | |
| Crude model | Ref. | -0.87 (-2.09~0.35), P=0.148 | -0.76 (-2.03~ 0.52), P=0.219 | -0.88 (-2.35~ 0.59), P=0.216 | 0.246 |
| Model1 | Ref. | -0.72 (-1.66~0.23), P=0.124 | -0.88 (-1.81~ 0.05), P=0.06 | -0.76 (-1.95~ 0.43), P=0.186 | 0.148 |
| Model2 | Ref. | -0.70 (-1.97~ 0.56), P=0.197 | -0.89 (-2.07~ 0.28), P=0.103 | -0.80 (-2.34~ 0.75, P=0.225 | 0.175 |
| female | | | | | |
| Crude model | Ref. | -0.73 (-1.46~-0.002), P=0.049 | -1.08 (-1.75~ -0.41), P=0.004 | -1.07 (-1.81~ -0.03), P=0.008 | 0.009 |
| Model1 | Ref. | -0.82 (-1.46~-0.18), P=0.017 | -1.44 (-2.09~ -0.78), P<0.001 | -1.48 (-2.20~ -0.76), P=0.001 | <0.001 |
| Model2 | Ref. | -0.79 (-1.57~ -0.01), P=0.048 | -1.27 (-2.20~ -0.34), P=0.019 | -1.25 (-2.16~ -0.33, P=0.020 | 0.008 |

Note: Crude model: no covariates were adjusted; Model 1: Adjusted for age and body mass index; Model 2: Adjusted for variables in Model 1 plus race, education and poverty.

Abbreviations: OBS, oxidation balance score; AAC, abdominal aortic calcification; Q1~Q4: the first to fourth quantiles of the oxidation balance score.
