# Supplementary material for: Associations between oxidation balance score and abdominal aortic calcification, and the mediating role of glycohemoglobin: a nationally representative cross-sectional study from NHANES
Source: Front Nutr. 2025 Jan 20;12:1469449. doi: 10.3389/fnut.2025.1469449 (PMC11792858; doi:10.3389/fnut.2025.1469449)
Supplement: Supplementary file 2 [file Table_2.docx]

**Supplemental Table S2. Weighted linear regression between AAC score and OBS quartile in premenopausal women or postmenopausal women**

| Variables | OBS quartile, [Coefficient (95%CI), P] | | | | P-trend |
| --- | --- | --- | --- | --- | --- |
|  | Q1 | Q2 | Q3 | Q4 |  |
| Premenopausal women | | | | | |
| Crude model | Ref. | -0.05 (-0.51~0.42), P=0.835 | -0.25 (-0.61~ 0.10), P=0.150 | -0.03 (-0.46~ 0.51), P=0.901 | 0.991 |
| Model1 | Ref. | -0.03 (-0.51~0.46), P=0.910 | -0.22 (-0.63~ 0.19), P=0.259 | -0.04 (-0.47~ 0.56), P=0.859 | 0.930 |
| Model2 | Ref. | -0.01 (-0.66~ 0.63), P=0.964 | -0.22 (-0.75~ 0.31), P=0.317 | -0.02 (-0.65~ 0.70, P=0.932 | 0.993 |
| Postmenopausal women | | | | | |
| Crude model | Ref. | -0.96 (-1.84~-0.07), P=0.036 | -1.37 (-2.37~ -0.37), P=0.012 | -1.39 (-2.39~ -0.39), P=0.011 | 0.010 |
| Model1 | Ref. | -1.02 (-1.75~-0.28), P=0.012 | -1.76 (-2.54~ -0.98), P<0.001 | -2.14 (-3.05~ -1.24), P<0.001 | <0.001 |
| Model2 | Ref. | -0.95 (-1.81~ -0.10), P=0.037 | -1.54 (-2.64~ 0.45), P=0.017 | -1.83 (-2.98~ -0.67, P=0.012 | 0.003 |

Note: Crude model: no covariates were adjusted; Model 1: Adjusted for age and body mass index;Model 2: Adjusted for variables in Model 1 plus race, education and poverty.

Abbreviations: OBS, oxidation balance score; AAC, abdominal aortic calcification; Q1~Q4: the first to fourth quantiles of the oxidation balance score.
