# Supplementary material for: Associations between oxidation balance score and abdominal aortic calcification, and the mediating role of glycohemoglobin: a nationally representative cross-sectional study from NHANES
Source: Front Nutr. 2025 Jan 20;12:1469449. doi: 10.3389/fnut.2025.1469449 (PMC11792858; doi:10.3389/fnut.2025.1469449)
Supplement: Supplementary file 3 [file Table_3.docx]

**Supplemental Table S3. Two-by-two weighted linear regression between the OBS, glycohemoglobin and AAC**

| Variable | Coeffcient | 95%CI | P |
| --- | --- | --- | --- |
| linear regression ^1^ | -0.01 | -0.015~-0.004 | 0.003 |
| linear regression ^2^ | 0.57 | 0.33~0.80 | <0.001 |

Note: linear regression^1^ represents a weighted linear regression between the oxidative balance score and glycohemoglobin; linear regression^2^ represents a weighted linear regression between glycohemoglobin and AAC.

Abbreviations: OBS, oxidation balance score; AAC, abdominal aortic calcification.
